# The effects of a home-based resistance training programme on body composition and muscle function during weight loss in people living with overweight or obesity: a randomised controlled pilot trial

**DOI:** 10.1186/s12986-025-00986-1

**Published:** 2025-08-04

**Authors:** Ahmad Binmahfoz, Lynsey Johnston, Emma Dunning, Cindy M. Gray, Stuart R. Gray

**Affiliations:** 1 School of Cardiovascular and Metabolic Health, University of Glasgow, Glasgow, UK; 2 Department of Public Health, College of Health Sciences at Al-Leith, Umm Al-Qura University, Al-Leith, Kingdom of Saudi Arabia; 3 School of Social and Political Sciences, University of Glasgow, Glasgow, UK; 4 Institute of Sports Science and Innovation, Lithuanian Sports University, Kaunus, Lithuania

**Keywords:** Obesity, Overweight, Resistance exercise, Weight loss

## Abstract

**Background:**

Obesity continues to grow as a public health concern and although dietary interventions can be effective at reducing body mass and improving cardiovascular risk factors, they also result in undesirable losses of lean tissue. The aim of this randomised controlled pilot trial was to investigate the effects of a home-based resistance training exercise programme on body composition and muscle function in people living with overweight or obesity undergoing dietary weight loss.

**Methods:**

Participants (*n* = 48) from Glasgow were randomly assigned to either a diet-induced weight loss group (WL) or a diet plus home-based resistance training exercise group (RT + WL) for 12-weeks. Body composition, muscle strength, and physical function were assessed at baseline and post-intervention.

**Results:**

There was no effect of the resistance exercise training programme (all *p* > 0.05) on body composition including body mass index, total body mass, fat mass, fat free mass or muscle thickness during weight loss. However, the resistance training group showed improvements in muscle and physical function compared to the weight loss only group, resulting in higher grip strength (RT + WL: Δ2.65, 95% CI: 0.44, 4.86; WL: Δ-0.26, 95% CI: -2.04, 1.51:*p* = 0.046), maximal voluntary contraction force (RT + WL:Δ23.61, 95% CI: 3.39, 43.84 WL: Δ-11.95, 95% CI: -35.37, 11.48;*p* = 0.019), and sit-to-stand test scores (RT + WL:Δ5.9, 95% CI: 4.27, 7.53 WL: Δ1.47, 95% CI: 0.13, 2.82; *p* < 0.001).

**Conclusions:**

These findings suggest that incorporating home-based resistance training into weight loss programmes can preserve, or even enhance, muscle function without negatively impacting the effectiveness of dietary weight loss interventions, highlighting its potential to mitigate muscle function losses during weight loss in people living with overweight or obese.

**Trial registration:**

Name of the registry: ClinicalTrials.gov. The registration number: NCT05702840. Date of Registry: 18/01/2023. The registration title: EXerCise wEight Loss (EXCEL).

## Introduction

Obesity continues to grow as a public health concern and is associated with an increased risk of morbidity and mortality, and greater health and social care costs [1]. For example, obesity increases the risk of a range of chronic diseases, such as hypertension, type 2 diabetes, coronary heart disease, dyslipidaemia and certain cancers [2]. The prevalence of obesity continues to rise across the world, especially in the Middle East, Central and Eastern Europe and North America, with the global burden highest in adults (between 45 and 59 years of age) and women [3, 4]. Dietary interventions are a mainstay of the treatment of obesity, and a recent systematic review and meta-analysis has shown they result in significant weight loss of around 4–5 kg on average [5]. This level of weight loss results in improvements in cardiovascular risk factors such as blood pressure, low density lipoprotein (LDL) and high-density lipoprotein (HDL) cholesterol and glycaemic control [5]. Furthermore, larger levels of weight loss (~ 10 kg) via more intense dietary intervention, have been shown to result in remission of diabetes in almost half of participants [6].

Whilst these benefits are a major positive, one of the less desirable consequences of weight loss is the concomitant loss of lean tissue (which is a marker of muscle mass). Indeed around ∼20–30% of weight lost is lean tissue, with a recent meta-analysis showing that weight loss is associated with a loss of lean body mass of ~ 1.3 kg on average [7–9]. This is important as skeletal muscle has both functional and metabolic roles [10], with low muscle mass/strength recognised as a contributing factor to cardiometabolic and other obesity-related diseases [11] and as being associated with higher mortality and morbidity [7]. Therefore, although muscle mass and strength are generally higher in people with overweight/obesity [12], to maximise the benefits of weight loss it is optimal to retain muscle mass and strength as much as possible, and strategies to maintain lean tissue/muscle mass during weight loss are needed.

The most effective method to increase or maintain muscle mass is resistance exercise [13] which has been shown to not only increase muscle mass and strength but also to improve blood lipids and glycaemic control, reduce blood pressure and increase cardiorespiratory fitness in a variety of populations [14, 15]. There is also evidence that during weight loss, resistance exercise can attenuate the decline in fat free mass, augment fat loss and increase muscle strength [16, 17]. Thus far, studies of resistance exercise interventions have been primarily in supervised settings using traditional weight training machines or free weights, which can limit accessibility and thus scaling up.

The number of people regularly taking part in resistance exercise is, perhaps unsurprisingly, low [18] and several studies have reported barriers. For example, research with college women found that barriers included perceived lack of time, not feeling comfortable in the gym, as well as lack of knowledge regarding the use of free weights and other forms of resistance exercise [19, 20]. A systematic review reported that barriers to participation in resistance exercise for older adults include safety, fear, fatigue, health concerns, pain, and lack of social support [21, 22]. Another recent study found that 68% of adults with obesity reported difficulty accessing gym facilities and discomfort in public exercise settings [23]. It has also been recently demonstrated that the COVID-19 pandemic has further emphasised the need for accessible, home-based exercise options [24, 25].

One potential strategy to increase accessibility is to develop interventions based on simple resistance exercises with minimal equipment that can be carried out at home. It has been shown, in a variety of populations, that home-based resistance exercises can have positive effects on body weight/composition outcomes and markers of cardiometabolic health [26] alongside increases in muscle strength, physical functioning [27] and functional mobility [28]. However, the impact of home-based resistance exercise on body composition and muscle strength during weight loss in people living with overweight or obesity has not yet been explored. The aim of the current pilot study, therefore, is to investigate the effects of home-based resistance exercise programme on changes in body composition and strength during weight loss in people living with overweight or obesity. This data will inform the development of a larger fully powered randomised controlled trial.

## Methods

This study is reported following the Consolidated Standards of Reporting Trials (CONSORT) Checklist [29] and registered on ClinicalTrials.gov (registration number: NCT05702840, date of registration January 18, 2023).

### Study design

This study was a 12-week, parallel group pilot randomised controlled trial with participants randomly assigned (1:1) to either (1) diet induced weight loss (WL) or (2) diet induced weight loss plus home-based resistance training (RT + WL). The study was approved by the College of Medical Veterinary and Life Sciences Ethics Committee at the University of Glasgow and all participants provided written informed consent.

### Sample size

The current study is a pilot study and so no formal sample size calculation was carried out. We aimed to recruit 50 participants, which is within the recommended range of sample size for pilot studies [30] and would allow us to detect a 0.8 SD difference in outcomes (power 80%, alpha = 0.05).

### Participants

Participants were recruited from in and around Glasgow by leaving posters and/or flyers at various public places and advertising the study online on social media platforms, such as Facebook and X, from February 2023 to December 2023. Inclusion criteria were: Body mass index (BMI) ≥ 25 kg/m2; aged 18–65 years; and passing the Physical Activity Readiness Questionnaire (PAR-Q+). Exclusion criteria were: currently taking part in more than 1.5 h of structured exercise per week; having recently (< 6 months) taken part in any resistance exercise training; taking any medications known to affect weight loss; being actively engaged in a weight loss programme; having lost more than 2 kg weight in the last 6 months; and any other reason that would limit ability to perform the exercises and outcome measurements safely.

### Randomisation

Randomisation was conducted upon completion of baseline measurements by an independent researcher using sealed envelopes [31] with participants randomised to either the RT + WL group or WL group.

### Interventions

#### Weight loss

All participants were provided access to the Weight Watchers weight loss programme for a 12-week period. Weight Watchers is a commercially available programme [32], and participants set an initial goal to lose 5 kg of body mass over the 12 weeks. If 5 kg weight loss was achieved, then the participant could choose further weight loss goals. The Weight Watchers programme works on the basis of an individualised points plan that could then be used by the participant to select foods/meals to consume throughout the day.

### Home based resistance training

Participants in the RT + WL group also received a resistance exercise booklet containing instructions for exercises and links to demonstration videos. A demonstration and explanation of the exercises was given face-to-face at the beginning of the intervention, alongside a discussion of the principles of the programme, including starting level and progression. We asked participants to perform the resistance exercises three times a week throughout the 12-week period. Participants were asked to perform three sets of each exercise, with the goal of reaching a Rating of Perceived Exertion (RPE) of between 8 and 10 (on a scale of 1–10, where 10 represents maximal effort). In order to build up intensity slowly, participants were asked to target a lower RPE of 4–6 during the first week [33]. The exercises included were press-ups, band lateral raises, band seated low rows, squats, lunges and calf raises, with different levels depending on participants’ baseline abilities, and to allow progression.

### Outcomes measures

Prior to the start of the intervention, height, blood pressure and heart rate were measured. At baseline and after the 12-week intervention period the following outcomes were measured: body mass, body composition, muscle strength and physical function. Participants were asked to avoid strenuous exercise prior to the measurements, which were performed at the same time of day at each timepoint.

### Body mass and composition

BMI and body composition were measured using scales and a bioelectrical impedance device to quantify fat mass and fat free mass [34]. We also measured vastus lateralis muscle thickness, as a measure of muscle size, using ultrasound [35].

### Muscle strength

Knee extensor maximal torque during a maximum voluntary contraction (MVC) was measured with participants strapped in a chair with their legs at a 90-degree angle. A strap was placed around the right ankle which was connected to a force transducer. Participants were asked to contract maximally with the leg fixed in position for 10 s. Participants performed three contractions, with 60 s rest between contractions, and if the 3rd contraction was > 10% of the 2nd contraction, then a 4th contraction was performed. The highest value was used in the analysis. Grip strength was measured using a Jamar dynamometer, with participants asked to perform three maximal contractions in each hand. The highest value was used in the analysis.

### Physical function

A 30-second sit-to-stand test (STS) was used to assess physical function. Participants were asked to sit in a chair with their hands crossed across their shoulders, rise to a full standing position, then sit back down again and repeat this for 30 s as quickly as they could. The number of full repetitions was recorded and used for analysis.

### Statistical analysis

Descriptive baseline characteristics of both groups are presented as means and standard deviations (SD). Using SPSS 29.0.1.0, differences between the groups in outcomes at 12 weeks were assessed using analysis of covariance (one-way ACNOVA) with baseline outcome included as a covariate. Prior to data analysis, tests of normality by Kolmogorov-Smirnov and assumptions for conducting one-way ANCOVA were assessed, with data meeting all necessary assumptions [36]. A p value of < 0.05 was used for statistical significance.

## Results

**Fig. 1:**
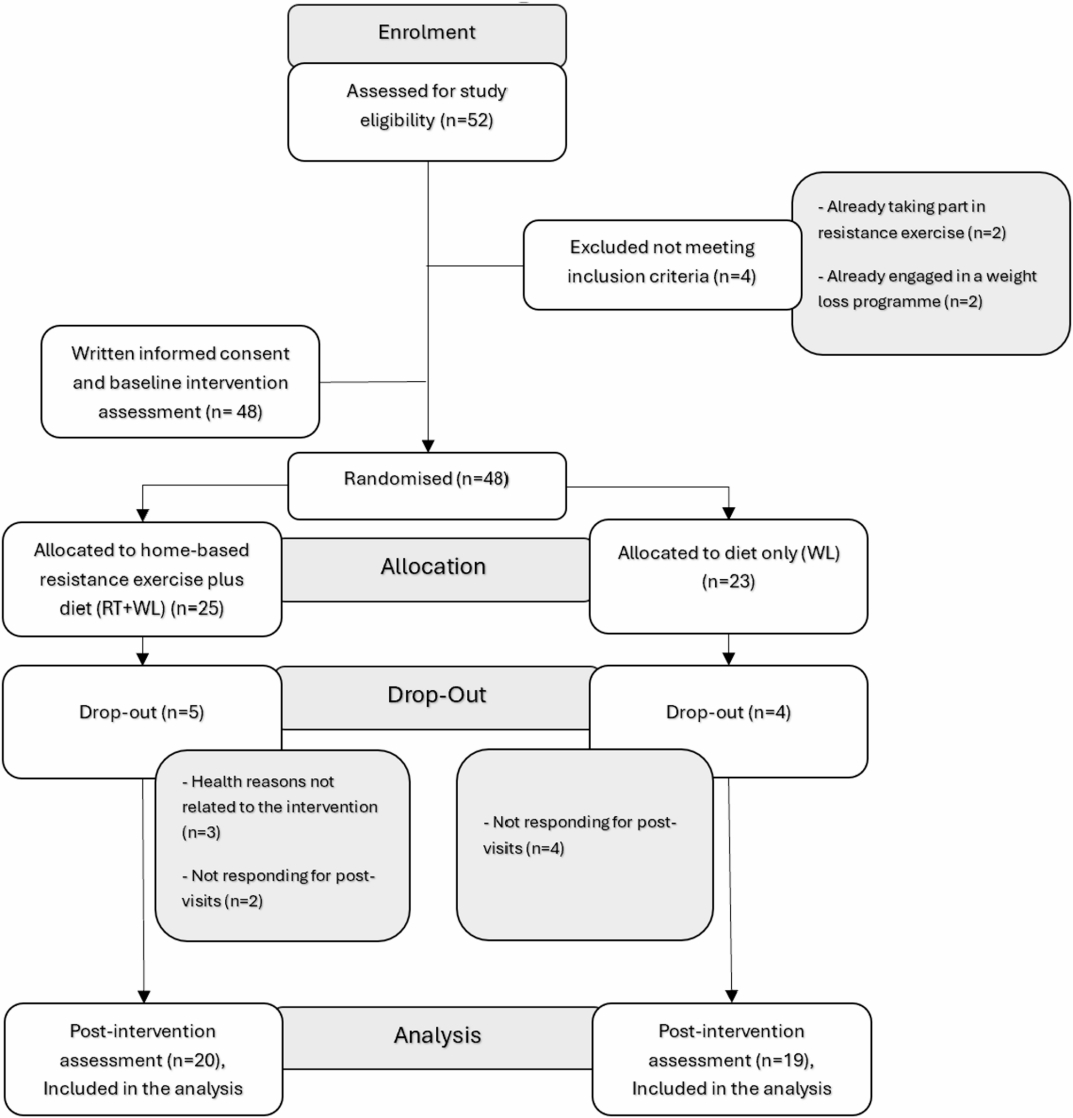
CONSORT diagram updated

From February to December 2023, a total of 48 participants were recruited and randomised. Of these, 39 individuals successfully completed the study (26 males and 13 females) (Fig. 1 provides the CONSORT diagram of participant recruitment, randomisation, dropout and analysis).

Baseline demographics characteristics for all participants, completers and non-completers, are provided in Table 1. There were no clear differences between the groups, although the WL group were slightly younger and had a slightly higher proportion of female participants. Comparing completers and non-completers the majority of non-completers were female.

**Table 1:** Baseline demographics characteristics. Data are presented as mean (SD)

	RT + WL group (*n* = 20) completers			RT + WL group (*n* = 5) non-completers			RT + WL group (*n* = 25) all participants			WL group (*n* = 19) completers			WL group (*n* = 4) non-completers			WL group (*n* = 23) all participants		
Age	42 (11.76)			32 (12.55)			40 (12.15)			37 (8.74)			36 (16.47)			37 (9.99)		
*Sex	Male	(*n* = 15)	(75%)	Male	(*n* = 1)	(20%)	Male	(*n* = 16)	(64%)	Male	(*n* = 11)	(58%)	Male	(*n* = 0)	(0%)	Male	(*n* = 11)	(48%)
	Female	(*n* = 5)	(25%)	Female	(*n* = 4)	(80%)	Female	(*n* = 9)	(36%)	Female	(*n* = 8)	(42%)	Female	(*n* = 4)	(100%)	Female	(*n* = 12)	(52%)
Height (cm)	169 (8.14)			171 (12.07)			169 (8.79)			170 (10.13)			165 (5.598)			170 (9.65)		
Systolic blood pressure (mmHg)	125 (13.83)			135 (15.42)			127 (14.57)			129 (16.04)			122 (16.87)			128 (16.03)		
Diastolic blood pressure (mmHg)	77 (8.96)			87 (6.06)			79 (9.39)			79 (9.88)			81 (8.699)			80 (9.53)		
Heart rate	77 (15.64)			85 (10.26)			79 (14.77)			77 (13.74)			86 (17.269)			79 (14.34)		
Weight (kg)	85.2 (17.5)			96.3 (29.1)			87 (20.1)			86.9 (14.5)			77 (13.1)			85.2 (14.5)		
^a^ BMI (kg/m2)	29.9 (6.2)			33.1 (9.9)			30.5 (6.9)			29.8 (3.6)			28.1 (2.9)			29.5 (3.5)		
Fat mass (kg)	25.4 (13.4)			39.8 (23.7)			28.3 (16.4)			28.7 (9.6)			28.1 (10.1)			28.6 (9.4)		
Fat percentage (%)	29 (10.2)			39 (11.9)			31 (11.1)			32.9 (9)			35.8 (6.9)			33.5 (8.6)		
Fat free mass (kg)	59.8 (10.8)			56.6 (9.6)			59.2 (10.4)			58.2 (12.5)			48.9 (5.8)			56.6 (12)		
Muscle thickness (mm)	24.4 (3.4)			23.9 (2.6)			24.4 (3.2)			25.7 (2.9)			27.2 (4.9)			25.9 (3.3)		
Knee extensor maximal torque (N)	491.3 (79.8)			454.4 (82.6)			480.1 (80.9)			492.8 (75.1)			436.6 (107.4)			483 (81.7)		
Grip strength (kg)	37 (8.8)			36.8 (13.3)			36.7 (9.4)			38.2 (9)			25.5 (9.6)			35.9 (10.1)		
^b^ STS	15.9 (3.9)			17 (4.2)			16 (3.9)			16 (2.7)			16.8 (2.2)			16.1 (2.6)		

*Data are presented as n = number of participants (%)

^a^BMI: Body mass index

^b^STS: 30-second sit-to-stand test

### Post-intervention outcomes

After the 12-week intervention period, there was no difference in BMI (*p* = 0.642), body mass (*p* = 0.822), fat mass (*p* = 0.729), fat percentage (*p* = 0.797), fat free mass (*p* = 0.739) or muscle thickness (*p* = 0.598) between the RT + WL and WL groups (Table 2). The ANCOVA revealed a significant difference in grip strength between the RT + WL and WL groups (*p* = 0.046), with a higher grip strength in the RT + WL compared to the WL group at 12 weeks. Resistance exercise training during weight loss also resulted in a higher knee extensor maximal torque in the RT + WL, compared to the WL, group at 12 weeks (*p* = 0.019). Similarly at 12 weeks, there was a higher STS in the RT + WL, compared to the WL group (*p* < 0.001). Muscle function and strength data are shown in Table 3.

**Table 2:** Differences in body weight and composition between groups before and after 12 weeks. Data are presented as mean (SD)

Outcome Variable	RT + WL group (*n* = 20)			WL group (*n* = 19)			^a^ Post intervention mean difference (ANCOVA)	95% Confidence Interval for Difference		^b^ *p* value (ANCOVA)
	Pre	Post	*Change (95%CI)	Pre	Post	*Change (95%CI)		Lower bound	Upper bound	
^c^ BMI(kg/m2)	29.9 (6.2)	29 (5.6)	-0.9 (-1.33, -0.41)	29.8 (3.6)	28.8 (3.1)	-1 (-1.61, -0.39)	0.14	− 0.47	0.75	0.642
Weight (kg)	85.2 (17.5)	82.8 (15.8)	-2.4 (-3.77, -1.13)	86.9 (14.5)	84.1 (13.9)	-2.8 (-4.63, -1.03)	0.22	-1.71	2.15	0.822
Fat mass (kg)	25.4 (13.4)	24.2 (12.4)	-1.2 (-2.48, 0.02)	28.7 (9.6)	26.8 (8.3)	-1.9 (-3.59, -0.29)	0.31	-1.49	2.11	0.729
Fat percentage (%)	29 (10.2)	28.4 (9.8)	-0.6 (-1.87, 0.68)	33 (9)	31.8 (8.4)	-1.2 (-2.44, 0.11)	0.22	-1.49	1.94	0.797
Fat free mass (kg)	59.8 (10.8)	58.6 (9.6)	-1.2 (-2.41, -0.03)	58.2 (12.5)	57.3 (11.9)	-0.9 (-1.62, -0.14)	− 0.21	-1.45	1.04	0.739
Muscle thickness (mm)	24.4 (3.4)	24.3 (3.2)	-0.1 (-1.3, 1.15)	25.7 (2.9)	25 (3.7)	-0.7 (-1.75, 0.27)	0.41	-1.14	1.95	0.598

*Change data are presented as mean (95%) CI (lower, upper)

a. Adjusted mean differences for the post-outcome variable when controlling the pre-outcome variable

b. Significant difference for the ANCOVA test

c. BMI: Body mass index

**Table 3:** Differences in muscle function and strength between groups before and after 12 weeks. Data are presented as mean (SD)

Outcome Variable	RT + WL group (*n* = 20)			WL group (*n* = 19)			^a^ Post intervention mean difference (ANCOVA)	95% Confidence Interval for Difference		^b^ *p* value (ANCOVA)
	Pre	Post	*Change (95%CI)	Pre	Post	*Change (95%CI)		Lower bound	Upper bound	
Knee extensor maximal torque (N)	491.3 (79.8)	514.9 (72.2)	23.6 (3.39, 43.84)	492.8 (75.1)	480.8 (86.9)	-12 (-35.37, 11.48)	35.35	6.05	64.65	0.019
Grip strength (kg)	37 (8.8)	39.7 (9.5)	2.7 (0.44, 4.86)	38.1 (9)	37.9 (8.8)	-0.2 (-2.04, 1.51)	2.82	0.05	5.58	0.046
^c^ STS	15.9 (3.9)	21.8 (4.9)	5.9 (4.27, 7.53)	16 (2.7)	17.5 (3.9)	1.5 (0.13, 2.82)	4.42	2.35	6.49	< 0.001

*Change data are presented as mean (95%) CI (lower, upper)

a. Adjusted mean differences for the post-outcome variable when controlling the pre-outcome variable

b. Significant difference for the ANCOVA test

c. 30-second sit-to-stand test

## Discussion

The current pilot study aimed to investigate the effects of a home-based resistance exercise training programme on body composition, and muscle function and strength during weight loss in adults living with overweight or obesity. We found that home-based resistance exercise during weight loss had no effect on body composition, including body mass index, body mass, fat mass, fat free mass, or muscle thickness, but did lead to improvements in muscle strength and function, including grip strength, knee extensor maximal torque and sit-to-stand performance. This pilot study provides preliminary efficacy data which can inform the design of a larger, fully powered randomised controlled trial investigating the effect home-based resistance exercise during weight loss.

Currently, there is limited evidence available on the effects of home-based resistance exercise training during weight loss in adults living with overweight or obesity. Although our data indicates that this is not sufficient to preserve the loss of fat free mass, these losses were relatively small in the current pilot study. This may have limited our ability to detect any effect of home based resistance exercise on fat free mass or muscle thickness and further work applying this intervention during more extreme loss of fat free mass, for example with the use of total diet replacement or weight loss medications such as the glucagon-like peptide 1 (GLP-1) agonists, are warranted [37]. In addition, we employed bioelectrical impedance analysis (BIA) to measure fat free mass and ultrasound to measure vastus lateral thickness, which are not the gold standard methods for assessment of muscle mass. Moreover, the relatively low sample size in this pilot work, it is likely we did not have the sensitivity or statistical power to detect differences in either fat free mass or muscle thickness.

It is also possible that we did not see effects on body composition with our intervention due to the resistance exercises being home-based and unsupervised. Indeed previous studies of supervised resistance exercise during weight loss demonstrated increases in fat free mass [16, 38], decreases in fat mass [39, 40] and improved muscle strength [16, 40]. Furthermore, a recent meta-analysis in older adults with obesity found that supervised resistance exercise training attenuated the loss of lean mass during weight loss (mean difference: 0.8 kg [95% CI: 0.4–1.3 kg]) [41]. Also, a recent systematic review and meta-analysis concluded that supervised resistance training combined with caloric restriction was effective for lowering body fat percentage (by 2.9–4.7%) in people living with overweight or obesity during weight loss [42]. As mentioned, these studies involved supervised, facility-based exercise interventions, and this may have provided a higher training stimulus compared to our home-based programme for the retention of fat free mass [43–46]. There is, to our knowledge, only one relevant previous study of home-based resistance exercise, although this was in the weight maintenance phase which, similar to the current study, found that such exercise had no effect on fat free mass [47].

Although the current study did not find any effect of the intervention on measures of body composition, we did find increases in measures of muscle function, including grip strength, knee extensor maximal torque and sit-to-stand performance, indicative of an increase in muscle quality. These results are consistent with previous studies of supervised resistance exercise training during weight loss which found that muscle strength was increased. For example, a recent systematic review and meta-analysis found that supervised resistance exercise can improve muscle strength (standardised mean difference: 1.79, 95% CI: 0.91–2.67) during weight loss in people living with obesity or overweight [48]. Further research also observed improvement in strength with resistance exercise during weight loss, including handgrip strength (+ 1.2 ± 2.5 kg, *P* < 0.001) and knee extensor torque (+ 7.9 ± 19.1 N-m, *P* < 0.001) [40]. Other studies reported that including resistance exercise during dietary weight loss can help maintain muscle function [49] as assessed by a 4-minute walk test, a 6-minute walk test and sit-to-stand (STS) performance, and strength [16] measured by symptom-limited one-repetition maximum (1-RM). Furthermore, despite using different methods for assessing muscle strength such as eight-repetition maximum (8RM) [50] or one-repetition maximum (1RM) [51], studies in postmenopausal women living with overweight and obesity research have shown that supervised resistance exercise training has a positive impact on muscle strength during weight loss. Overall therefore, existing evidence supports the assertion that resistance exercise during weight loss can be beneficial in enhancing and improving overall muscle strength and physical function, with the current study demonstrating that this can be achieved by a home based simple and pragmatic intervention.

The observed improvements in muscular strength and function, despite no changes in body composition in our study, could be due to several reasons including neural adaptations and improved neuromuscular function that occur with resistance training. There is evidence that resistance training exercise induces neural adaptations, such as increased recruitment of motor units, firing frequency, and coordinated movements between muscles, which can enhance force production and increase strength independent of hypertrophic or body composition changes [52, 53]. There is also, during weight loss, research showing that a caloric restriction diet and the associated energy deficit may impair the muscle protein synthesis response to resistance exercise, limiting hypertrophic adaptations and the resources required to build new muscle tissue [7, 54, 55]. However, these factors may have a lesser impact on the neural adaptations related to strength improvements [56, 57].

The home-based nature of our intervention was designed to address several barriers to resistance exercise participation commonly cited in the literature, including accessibility, cost, time constraints and not feeling comfortable in the gym. However, we acknowledge that home-based programmes may present other challenges, such as reduced social support and lack of professional supervision, which could impact adherence and effectiveness. Future larger studies should formally assess adherence rates, participant satisfaction and barriers to completion to better understand the long term feasibility of this approach.

Among the key strengths of our study is that we used a randomised controlled design, which is considered the gold standard. We also employed a home-based resistance training programme to raise the ecological validity and real-world applicability of the findings. The current study provides preliminary evidence for the potential impacts of an accessible resistance exercise intervention, although formal feasibility assessment including adherence measurements and participants feedback would be required before being applied more widely. This needs to be tested in a larger scale, appropriately powered randomised controlled trial. However, before moving on with a larger scale trial, further investigation is needed to better replicate the benefits reported in supervised resistance training interventions within a home-based programme.

It is important to point out the limitations of our study. This pilot trial had a small sample size which means we did not have the statistical power to detect significant changes in all outcome measures. A key limitation is that, although participants in the resistance exercise group received exercise materials and instructions, adherence was not formally assessed, as we aimed to ensure the intervention was pragmatic and light touch for the participant. This may explain the absence of effects on body composition measures. Without objective measures of exercise compliance, we cannot determine whether participants consistently performed the prescribed exercises with adequate intensity and frequency to elicit the physiological adaptations necessary for body composition changes, although it is worth noting that changes in strength and function were observed– implying reasonable adherence to the exercise intervention. On top of this, the current study did not explore participant experiences. Together this limits our ability to comment on the long term feasibility of this intervention. Although participants were asked to follow a weight loss programme for diet, dietary intake was not strictly controlled or monitored and it is possible that dietary compliance and macronutrient composition could have affected the findings, particularly in terms of body composition changes.

Furthermore, our use of BIA to assess fat free mass represents a significant methodological limitation. BIA has lower precision compared to dual-energy X-ray absorptiometry (DXA), the gold standard for body composition assessment used in similar studies [58]. Within our lab the coefficient of variation for measurement of fat free mass is 1.3% (unpublished data), and previous work has reported a minimum detectable change in the order of 3.5–4 kg for fat free mass [59]. This may contribute to the lack of observed effect of the intervention in fat free mass and explain the substantial fat free mass loss observed in the RT + WL group, which is larger than expected. This finding likely reflects the limitations of BIA in detecting muscle preserving effects of resistance exercise, highlighting the need for more precise body composition assessment in future studies investigating similar interventions. Another limitation was a lack of control or assessment of protein intake during the intervention. The Weight Watchers programme encourages lean protein consumption through ZeroPoint foods but does not specify minimum protein targets in g/kg body weight, with intake on average ~ 0.9 g/kg/day [60]. It has been demonstrated that protein intake ≥ 1.2 g/kg body weight is critical for muscle mass preservation during weight loss in obese older adults [61], and it may be that protein intake was not sufficient to, alongside exercise, support maintenance of fat free mass during the intervention. Future studies should include formal protein intake assessment and potentially provide specific protein targets to optimise muscle preservation during weight loss interventions.

The improvements in muscular strength and physical function observed with resistance exercise training during weight loss have important clinical and functional implications [49, 62]. Preserving or increasing muscle strength and functional capacity can improve quality of life [63] and independence in everyday tasks, and lower the risk of falls and disabilities linked with ageing and obesity [64, 65]. Resistance exercise training has also been found to have major metabolic health benefits, such as enhanced insulin sensitivity and better glucose management, which are vital for controlling and avoiding type 2 diabetes and other metabolic disorders [66–68]. Although weight loss can sometimes decrease muscle strength due to the reduction in body mass [7], it often improves physical function by reducing the mechanical load on the body [69]. However, the further enhancement of muscle strength and physical function observed with resistance exercise training is still a positive outcome for long-term health, as it helps mitigate the potential loss of strength and enhances functional capacity beyond what is achieved through weight loss alone. Therefore, combining weight loss with a home-based resistance training programme seems to have further benefits for overall health and may be an effective way for people living with overweight and obesity to engage in an exercise intervention that is accessible and addresses several common barriers to resistance exercise participation, although adherence rates and long-term sustainability require further investigation. Regular physical activity is significantly challenging for this population, and this approach could facilitate adoption and maintenance of regular physical activity.

In conclusion, this pilot randomised controlled trial found that a home-based resistance training programme during weight loss in people living with overweight or obesity had no impact body composition measures such as BMI, body mass, fat mass, or fat free mass. However, improvements were found in grip strength, knee extensor maximal torque and sit-to-stand performance. Overall, this study highlights the potential value of incorporating home based resistance training into weight loss programmes for adults living with overweight or obesity, as it can assist in maintaining strength and physical capability.

## Data Availability

The datasets used and/or analysed during the current study are available from the corresponding author on reasonable request.

## Abbreviations

LDL: Low density lipoprotein
HDL: High-density lipoprotein
CONSORT: Consolidated Standards of Reporting Trials Checklist
WL: Diet induced weight loss
RT + WL: Diet induced weight loss plus home-based resistance training
BMI: Body mass index
PAR-Q+: Physical Activity Readiness Questionnaire
RPE: Rating of Perceived Exertion
MVC: Maximum voluntary contraction
STS: 30-second sit-to-stand test
SD: Standard deviations
GLP-1: Glucagon-like peptide 1
1-RM: Symptom-limited one-repetition maximum
8RM: Eight-repetition maximum
1RM: One-repetition maximum

## References

[1] Upadhyay J, Farr O, Perakakis N, Ghaly W, Mantzoros C. Obesity as a disease. Med Clin N Am. 2018;102(1):13–33. 29156181 10.1016/j.mcna.2017.08.004

[2] An R, Ji M, Zhang S. Global warming and obesity: a systematic review. Obes Rev. 2018;19(2):150–63. 28977817 10.1111/obr.12624

[3] James PT, Leach R, Kalamara E, Shayeghi M. The worldwide obesity epidemic. Obes (Silver Spring Md). 2001;9(11S):S228–33. 10.1038/oby.2001.123 11707546

[4] Siervo M, Montagnese C, Mathers JC, Soroka KR, Stephan BCM, Wells JCK. Sugar consumption and global prevalence of obesity and hypertension: an ecological analysis. Public Health Nutr. 2014;17(3):587–96. 23414749 10.1017/S1368980013000141 PMC10282320

[5] Ge L, Sadeghirad B, Ball GDC, da Costa BR, Hitchcock CL, Svendrovski A, et al. Comparison of dietary macronutrient patterns of 14 popular named dietary programmes for weight and cardiovascular risk factor reduction in adults: systematic review and network meta-analysis of randomised trials. BMJ (Online). 2020;369:m696–m. 10.1136/bmj.m696 PMC7190064 32238384

[6] Lean MEJ, Leslie WS, Barnes AC, Brosnahan N, Thom G, McCombie L, et al. Primary care-led weight management for remission of type 2 diabetes (DiRECT): an open-label, cluster-randomised trial. Lancet (British edition). 2018;391(10120):541–51. 10.1016/S0140-6736(17)33102-1 29221645

[7] Cava E, Yeat NC, Mittendorfer B. Preserving healthy muscle during weight loss. Advances in nutrition (Bethesda. Md). 2017;8(3):511–9. 10.3945/an.116.014506 PMC5421125 28507015

[8] Enriquez Guerrero A, San Mauro Martin I, Garicano Vilar E, Camina Martin MA. Effectiveness of an intermittent fasting diet versus continuous energy restriction on anthropometric measurements, body composition and lipid profile in overweight and obese adults: a meta-analysis. Eur J Clin Nutr. 2021;75(7):1024–39. 33293678 10.1038/s41430-020-00821-1

[9] Pellegrini M, Cioffi I, Evangelista A, Ponzo V, Goitre I, Ciccone G, et al. Effects of time-restricted feeding on body weight and metabolism. A systematic review and meta-analysis. Reviews Endocr Metabolic Disorders. 2019;21(1):17–33. 10.1007/s11154-019-09524-w 31808043

[10] Wolfe RR. Underappreciated role of muscle in health and disease. Am J Clin Nutr. 2006;84(3):475–82. 16960159 10.1093/ajcn/84.3.475

[11] Sajoux I, Lorenzo PM, Gomez-Arbelaez D, Angeles Zulet M, Abete I, Castro A, et al. Effect of a Very-Low-Calorie ketogenic diet on Circulating myokine levels compared with the effect of bariatric surgery or a Low-Calorie diet in patients with obesity. Nutrients. 2019;11(10):2368. 31590286 10.3390/nu11102368 PMC6835835

[12] Tomlinson DJ, Erskine RM, Morse CI, Winwood K, Onambélé-Pearson G. The impact of obesity on skeletal muscle strength and structure through adolescence to old age. Biogerontology. 2016;17:467–83. 26667010 10.1007/s10522-015-9626-4 PMC4889641

[13] Westcott WL. Resistance training is medicine: effects of strength training on health. Curr Sports Med Rep. 2012;11(4):209–16. 22777332 10.1249/JSR.0b013e31825dabb8

[14] Ashton RE, Tew GA, Aning JJ, Gilbert SE, Lewis L, Saxton JM. Effects of short-term, medium-term and long-term resistance exercise training on cardiometabolic health outcomes in adults: systematic review with meta-analysis. Br J Sports Med. 2020;54(6):341–8. 29934430 10.1136/bjsports-2017-098970

[15] Cornelissen VA, Fagard RH, Coeckelberghs E, Vanhees L. Impact of resistance training on blood pressure and other cardiovascular risk factors: A Meta-Analysis of randomized, controlled trials. Hypertension (Dallas, Tex 1979). 2011;58(5):950–8. 10.1161/HYPERTENSIONAHA.111.177071 21896934

[16] Avila JJ, Gutierres JA, Sheehy ME, Lofgren IE, Delmonico MJ. Effect of moderate intensity resistance training during weight loss on body composition and physical performance in overweight older adults. Eur J Appl Physiol. 2010;109(3):517–25. 20169360 10.1007/s00421-010-1387-9

[17] Hunter GR, Fisher G, Neumeier WH, Carter SJ, Plaisance EP. Exercise training and energy expenditure following weight loss. Med Sci Sports Exerc. 2015;47(9):1950–7. 25606816 10.1249/MSS.0000000000000622 PMC4508245

[18] Strain T, Fitzsimons C, Kelly P, Mutrie N. The forgotten guidelines: cross-sectional analysis of participation in muscle strengthening and balance & co-ordination activities by adults and older adults in Scotland. BMC Public Health. 2016;16:1–12. 27769211 10.1186/s12889-016-3774-6 PMC5073826

[19] Hurley KS, Flippin KJ, Blom LC, Bolin JE, Hoover DL, Judge LW. Practices, perceived benefits, and barriers to resistance training among women enrolled in college. Int J Exerc Sci. 2018;11(5):226. 29795737 10.70252/ZRMT3507 PMC5955292

[20] Peters NA, Schlaff RA, Knous JL, Baruth M. Barriers to resistance training among college-aged women. J Am Coll Health. 2019;67(1):4–9. 29652602 10.1080/07448481.2018.1462815

[21] Burton E, Farrier K, Lewin G, Pettigrew S, Hill A-M, Airey P, et al. Motivators and barriers for older people participating in resistance training: a systematic review. J Aging Phys Act. 2017;25(2):311–24. 27620535 10.1123/japa.2015-0289

[22] Cavill NA, Foster CEM. Enablers and barriers to older people’s participation in strength and balance activities: A review of reviews. J Frailty Sarcopenia Falls. 2018;3(2):105. 32300698 10.22540/JFSF-03-105 PMC7155318

[23] Schvey NA, Sbrocco T, Bakalar JL, Ress R, Barmine M, Gorlick J, et al. The experience of weight stigma among gym members with overweight and obesity. Stigma Health. 2017;2(4):292.

[24] Nyenhuis SM, Greiwe J, Zeiger JS, Nanda A, Cooke A. Exercise and fitness in the age of social distancing during the COVID-19 pandemic. J Allergy Clin Immunol Pract. 2020;8(7):2152. 32360185 10.1016/j.jaip.2020.04.039 PMC7187829

[25] Kaur H, Singh T, Arya YK, Mittal S. Physical fitness and exercise during the COVID-19 pandemic: A qualitative enquiry. Front Psychol. 2020;11:590172. 33250827 10.3389/fpsyg.2020.590172 PMC7673425

[26] Plotnikoff RC, Eves N, Jung M, Sigal RJ, Padwal R, Karunamuni N. Multicomponent, home-based resistance training for obese adults with type 2 diabetes: a randomized controlled trial. Int J Obes. 2010;34(12):1733–41. 10.1038/ijo.2010.109 20531348

[27] Mikesky AE, Topp R, Wigglesworth JK, Harsha DM, Edwards JE. Efficacy of a home-based training program for older adults using elastic tubing. Eur J Appl Physiol Occup Physiol. 1994;69:316–20. 7851367 10.1007/BF00392037

[28] Zion AS, De Meersman R, Diamond BE, Bloomfield DM. A home-based resistance-training program using elastic bands for elderly patients with orthostatic hypotension. Clin Auton Res. 2003;13:286–92. 12955554 10.1007/s10286-003-0117-3

[29] Schulz KF, Altman DG, Moher D, the CG. CONSORT 2010 statement: updated guidelines for reporting parallel group randomised trials. BMC Med. 2010;8(1):18. 20334633 10.1186/1741-7015-8-18 PMC2860339

[30] Whitehead AL, Julious SA, Cooper CL, Campbell MJ. Estimating the sample size for a pilot randomised trial to minimise the overall trial sample size for the external pilot and main trial for a continuous outcome variable. Stat Methods Med Res. 2016;25(3):1057–73. 26092476 10.1177/0962280215588241 PMC4876429

[31] Friedman LM, Furberg CD, DeMets DL, Reboussin DM, Granger CB. Fundamentals of clinical trials: Springer; 2015.

[32] Gudzune KA, Doshi RS, Mehta AK, Chaudhry ZW, Jacobs DK, Vakil RM, et al. Efficacy of commercial weight-loss programs: an updated systematic review. Ann Intern Med. 2015;162(7):501–12. 25844997 10.7326/M14-2238 PMC4446719

[33] Lagally KM, Robertson RJ. Construct validity of the OMNI resistance exercise scale. J Strength Conditioning Res. 2006;20(2):252–6. 10.1519/R-17224.1 16686549

[34] National Institutes of Health. Office of Medical Applications of Research. Bioelectrical impedance analysis in body composition measurement: National Institutes of health technology assessment conference statement, December 12–14, 1994: US Department of Health and Human Services, Public Health Service, National &#8230.

[35] Ismail AD, Alkhayl FFA, Wilson J, Johnston L, Gill JMR, Gray SR. The effect of short-duration resistance training on insulin sensitivity and muscle adaptations in overweight men. Exp Physiol. 2019;104(4):540–5. 30697876 10.1113/EP087435

[36] Huitema B. The analysis of covariance and alternatives: statistical methods for experiments, quasi-experiments, and single-case studies. Wiley; 2011.

[37] Conte C, Hall KD, Klein S. Is Weight Loss–Induced Muscle Mass Loss Clinically Relevant? JAMA. 2024. 10.1001/jama.2024.6586 38829659

[38] Campbell WW, Haub MD, Wolfe RR, Ferrando AA, Sullivan DH, Apolzan JW, et al. Resistance training preserves fat-free mass without impacting changes in protein metabolism after weight loss in older women. Obesity. 2009;17(7):1332–9. 19247271 10.1038/oby.2009.2 PMC4299870

[39] Miller T, Mull S, Aragon AA, Krieger J, Schoenfeld BJ. Resistance training combined with diet decreases body fat while preserving lean mass independent of resting metabolic rate: a randomized trial. Int J Sport Nutr Exerc Metab. 2018;28(1):46–54. 28871849 10.1123/ijsnem.2017-0221

[40] Straight CR, Dorfman LR, Cottell KE, Krol JM, Lofgren IE, Delmonico MJ. Effects of resistance training and dietary changes on physical function and body composition in overweight and obese older adults. J Phys Activity Health. 2012;9(6):875–83. 10.1123/jpah.9.6.875 21952180

[41] Sardeli AV, Komatsu TR, Mori MA, Gáspari AF, Chacon-Mikahil MPT. Resistance training prevents muscle loss induced by caloric restriction in obese elderly individuals: a systematic review and meta-analysis. Nutrients. 2018;10(4):423. 29596307 10.3390/nu10040423 PMC5946208

[42] Lopez P, Taaffe DR, Galvão DA, Newton RU, Nonemacher ER, Wendt VM, et al. Resistance training effectiveness on body composition and body weight outcomes in individuals with overweight and obesity across the lifespan: A systematic review and meta-analysis. Obes Rev. 2022;23(5):e13428. 35191588 10.1111/obr.13428 PMC9285060

[43] Coleman M, Burke R, Benavente C, Piñero A, Augustin F, Maldonado J, et al. Supervision during resistance training positively influences muscular adaptations in resistance-trained individuals. J Sports Sci. 2023;41(12):1207–17. 37789670 10.1080/02640414.2023.2261090

[44] Hunter GR, Byrne NM, Sirikul B, Fernández JR, Zuckerman PA, Darnell BE, et al. Resistance training conserves fat-free mass and resting energy expenditure following weight loss. Obesity. 2008;16(5):1045–51. 18356845 10.1038/oby.2008.38

[45] Fisher J, Steele J, Wolf M, Korakakis PA, Smith D, Giessing J. The role of supervision in resistance training; an exploratory systematic review and meta-analysis. Int J Strength Conditioning. 2022;2(1).

[46] Hurst C, Robinson SM, Witham MD, Dodds RM, Granic A, Buckland C, et al. Resistance exercise as a treatment for sarcopenia: prescription and delivery. Age Ageing. 2022;51(2):afac003. 35150587 10.1093/ageing/afac003 PMC8840798

[47] Dunstan DW, Daly RM, Owen N, Jolley D, Vulikh E, Shaw J, et al. Home-based resistance training is not sufficient to maintain improved glycemic control following supervised training in older individuals with type 2 diabetes. Diabetes Care. 2005;28(1):3–9. 15616225 10.2337/diacare.28.1.3

[48] Liu X, Gao Y, Lu J, Ma Q, Shi Y, Liu J, et al. Effects of different resistance exercise forms on body composition and muscle strength in overweight and/or obese individuals: a systematic review and meta-analysis. Front Physiol. 2022;12:791999. 35250604 10.3389/fphys.2021.791999 PMC8895240

[49] Orange ST, Madden LA, Vince RV. Resistance training leads to large improvements in strength and moderate improvements in physical function in adults who are overweight or obese: a systematic review. J Physiotherapy. 2020;66(4):214–24. 10.1016/j.jphys.2020.09.009 33069607

[50] Figueroa A, Vicil F, Sanchez-Gonzalez MA, Wong A, Ormsbee MJ, Hooshmand S, et al. Effects of diet and/or Low-Intensity resistance exercise training on arterial stiffness, adiposity, and lean mass in obese postmenopausal women. Am J Hypertens. 2013;26(3):416–23. 23382493 10.1093/ajh/hps050

[51] Hintze LJ, Messier V, Lavoie M-E, Brochu M, Lavoie J-M, Prud’homme D, et al. A one-year resistance training program following weight loss has no significant impact on body composition and energy expenditure in postmenopausal women living with overweight and obesity. Physiol Behav. 2018;189:99–106. 29549030 10.1016/j.physbeh.2018.03.014

[52] Folland JP, Williams AG. Morphological and neurological contributions to increased strength. Sports Med. 2007;37:145–68. 17241104 10.2165/00007256-200737020-00004

[53] Škarabot J, Brownstein CG, Casolo A, Del Vecchio A, Ansdell P. The knowns and unknowns of neural adaptations to resistance training. Eur J Appl Physiol. 2021;121:675–85. 33355714 10.1007/s00421-020-04567-3 PMC7892509

[54] Hector AJ, Marcotte GR, Churchward-Venne TA, Murphy CH, Breen L, von Allmen M, et al. Whey protein supplementation preserves postprandial myofibrillar protein synthesis during short-term energy restriction in overweight and obese adults. J Nutr. 2015;145(2):246–52. 25644344 10.3945/jn.114.200832

[55] Murphy C, Koehler K. Caloric restriction induces anabolic resistance to resistance exercise. Eur J Appl Physiol. 2020;120:1155–64. 32236752 10.1007/s00421-020-04354-0 PMC8233264

[56] Murlasits Z, Reed J. Muscular adaptations to periodized resistance-training in older adults. Sci Sports. 2020;35(4):216–22.

[57] Sale DG. Neural adaptation to resistance training. Med Sci Sports Exerc. 1988;20(5 Suppl):S135–45. 3057313 10.1249/00005768-198810001-00009

[58] Verreijen AM, Verlaan S, Engberink MF, Swinkels S, de Vogel-van den Bosch J, Weijs PJM. A high Whey protein–, leucine-, and vitamin D–enriched supplement preserves muscle mass during intentional weight loss in obese older adults: a double-blind randomized controlled trial. Am J Clin Nutr. 2015;101(2):279–86. 25646324 10.3945/ajcn.114.090290

[59] Koch B, Miller A, Glass NA, Owen E, Kirkpatrick T, Grossman R, et al. Reliability of multifrequency bioelectrical impedance analysis to quantify body composition in patients after musculoskeletal trauma. Iowa Orthop J. 2022;42(1):75. PMC9210418 35821931

[60] Palacios AM, Lee AM, Parker C, Watts CQ, Dickinson SL, Henschel B et al. Effectiveness of a digital weight management program on diet quality: A randomized controlled trial. Am J Clin Nutr. 2025.10.1016/j.ajcnut.2025.06.02440609748

[61] Weijs PJM, Wolfe RR. Exploration of the protein requirement during weight loss in obese older adults. Clin Nutr. 2016;35(2):394–8. 25788405 10.1016/j.clnu.2015.02.016

[62] Khodadad Kashi S, Mirzazadeh ZS, Saatchian V. A systematic review and meta-analysis of resistance training on quality of life, depression, muscle strength, and functional exercise capacity in older adults aged 60 years or more. Biol Res Nurs. 2023;25(1):88–106. 35968662 10.1177/10998004221120945

[63] Shaughnessy KA, Hackney KJ, Clark BC, Kraemer WJ, Terbizan DJ, Bailey RR, et al. A narrative review of handgrip strength and cognitive functioning: bringing a new characteristic to muscle memory. J Alzheimers Dis. 2020;73(4):1265–78. 31929158 10.3233/JAD-190856 PMC7063546

[64] Billot M, Calvani R, Urtamo A, Sánchez-Sánchez JL, Ciccolari-Micaldi C, Chang M et al. Preserving mobility in older adults with physical frailty and sarcopenia: opportunities, challenges, and recommendations for physical activity interventions. Clin Interv Aging. 2020: 15, 1675–90. 10.2147/CIA.S253535 PMC7508031 32982201

[65] Hillsdon M, Foster C. What are the health benefits of muscle and bone strengthening and balance activities across life stages and specific health outcomes? J Frailty Sarcopenia Falls. 2018;3(2):66. 32300695 10.22540/JFSF-03-066 PMC7155322

[66] Abou Sawan S, Nunes EA, Lim C, McKendry J, Phillips SM. The health benefits of resistance exercise: beyond hypertrophy and big weights. Exerc Sport Mov. 2023;1(1):e00001.

[67] Strasser B, Schobersberger W. Evidence for resistance training as a treatment therapy in obesity. J Obes. 2011;2011(1):482564. 20847892 10.1155/2011/482564 PMC2931407

[68] Pesta DH, Goncalves RLS, Madiraju AK, Strasser B, Sparks LM. Resistance training to improve type 2 diabetes: working toward a prescription for the future. Nutr Metabolism. 2017;14:1–10. 10.1186/s12986-017-0173-7 PMC5335813 28270856

[69] Santanasto AJ, Glynn NW, Newman MA, Taylor CA, Brooks MM, Goodpaster BH, et al. Impact of weight loss on physical function with changes in strength, muscle mass, and muscle fat infiltration in overweight to moderately obese older adults: a randomized clinical trial. J Obes. 2011;2011(1):516576. 20953373 10.1155/2011/516576 PMC2952914

